# Proteome Alterations in Cardiac Fibroblasts: Insights from Experimental Myocardial Infarction and Clinical Ischaemic Cardiomyopathy

**DOI:** 10.3390/ijms26083846

**Published:** 2025-04-18

**Authors:** Adam Russell-Hallinan, Claire Tonry, Lauren Kerrigan, Kevin Edgar, Patrick Collier, Ken McDonald, Mark Ledwidge, David Grieve, Narainrit Karuna, Chris Watson

**Affiliations:** 1 Wellcome-Wolfson Institute for Experimental Medicine, Queen’s University Belfast, Belfast BT9 7BL, Northern Ireland, UK; adamrh.ucd@gmail.com (A.R.-H.); claire.tonry@qub.ac.uk (C.T.); l.kerrigan@qub.ac.uk (L.K.); kevin.edgar@qub.ac.uk (K.E.); david.grieve@qub.ac.uk (D.G.); 2 Department of Cardiovascular Medicine, Cleveland Clinic, Cleveland, OH 44195, USA; 3 STOP-HF Unit, St Vincent’s University Hospital Healthcare Group, D04 T6F4 Dublin, Ireland; kenneth.mcdonald@ucd.ie (K.M.); mark.ledwidge@ucd.ie (M.L.); 4 UCD Conway Institute, School of Medicine, University College Dublin, D04 V1W8 Dublin, Ireland; 5 Department of Pharmaceutical Care, Faculty of Pharmacy, Chiang Mai University, Chiang Mai 50200, Thailand

**Keywords:** myocardial infarction, ischaemic heart disease, proteomics, TGF-β, cardiac remodelling, extracellular matrix

## Abstract

Ischaemic heart disease (IHD) is a chronic condition that can cause pathological cardiac remodelling and heart failure (HF). In this study, we sought to determine how cardiac fibroblasts were altered post-experimental myocardial infarction (MI). Female C57BL6 mice underwent experimental MI by permanent left coronary artery ligation. Cardiac fibroblasts were isolated from extracted heart tissue of experimental MI mice and subsequently treated with the pro-fibrotic cytokine, TGF-β, for 24 h and analysed using high throughput LC-MS/MS analysis. Findings were validated using mass spectrometry data generated from human left ventricular tissue analysis, which were collected from patients with ischaemic cardiomyopathy (ISCM) and age/sex-matched patients without clinical HF (NF). Proteomic analysis revealed significant protein expression changes in mouse cardiac fibroblasts after MI. These changes were most pronounced at 1 month post-MI, compared to earlier time points (3 days and 1 week). TGF-β treatment profoundly affected fibroblast cells extracted from MI mice, indicating a heightened sensitivity to pro-fibrotic factors after myocardial injury. Extracellular matrix (ECM) proteins significantly altered in MI fibroblasts following TGF-β treatment were significantly associated with cardiac remodelling. Notably, Lox was significantly changed in both isolated fibroblasts treated with TGF-β from experiment MI mice and human ISCM. Isolated cardiac fibroblasts from MI mice are more susceptible to developing pathogenic traits following TGF-β treatment than isolated fibroblasts from normal heart tissue. ECM proteins associated with these enhanced fibroblast activities and functions are evident. These altered proteins may play a functional role in MI-associated cardiac dysfunction.

## 1. Introduction

Myocardial infarction (MI) typically results from acute plaque rupture within the coronary arteries that supply oxygenated blood to the cardiac tissue and is a major cause of morbidity and mortality worldwide [1]. Acute MI has a high mortality rate and is difficult to diagnose and treat due to variable symptoms and sudden and unpredictable onset [2]. Although cardiac troponin is considered a useful clinical marker for myocardial injury, it takes time for cardiac troponin to be released into the bloodstream, which can lead to delayed diagnosis. Moreover, cardiac troponin can be elevated due to complications other than MI [2,3]. There is a need for additional biomarkers to help us better understand MI and the subsequent healing response, as well as a need for more effective therapeutic options to reverse the damage caused by MI. The majority of FDA-approved drugs for various diseases, including cardiovascular diseases, usually target human proteins [4]. Therefore, characterising pathophysiological changes to the cardiac proteome could reveal novel protein targets for therapeutic intervention.

The loss of perfusion to the affected area of cardiac tissue during MI causes cardiomyocyte necrosis, which cannot be compensated for due to the poor regenerative capacity of cardiomyocytes. Instead, tissue repair requires mobilisation and activation of fibroblasts to the injured site, which leads to deposition of fibrotic tissue to help maintain structural integrity; however, overproduction can result in cardiac dysfunction and heart failure (HF) [1,5,6,7]. Hence, cardiac fibroblasts are seen to play a prominent role in the evolution of ischaemic heart disease (IHD) to more advanced stages of HF [8,9]. Clinically, it would be beneficial to identify patients with perturbations in cardiac fibroblast activity and progressive fibrotic remodelling, as these patients could benefit from an intervention that targets fibroblast cells [1,10].

This study aimed to investigate the effect of MI on cardiac fibroblasts by comprehensively characterising resulting changes in the proteome of these cells from the different time points post-infarct induction (inflammatory phase, proliferative phase, and remodelling/maturation phase) along with examining the changes in response to pro-fibrotic activation by TGF-β. It is anticipated that these data could highlight potential therapeutic targets for better management of MI-induced complications and also reveal protein biomarkers of HF risk post-MI.

## 2. Results

### 2.1. Proteomic Characterisation of Myocardial Infarction Reveals Unique Proteomic Profile of Chronic Injury

**Figure 1:**
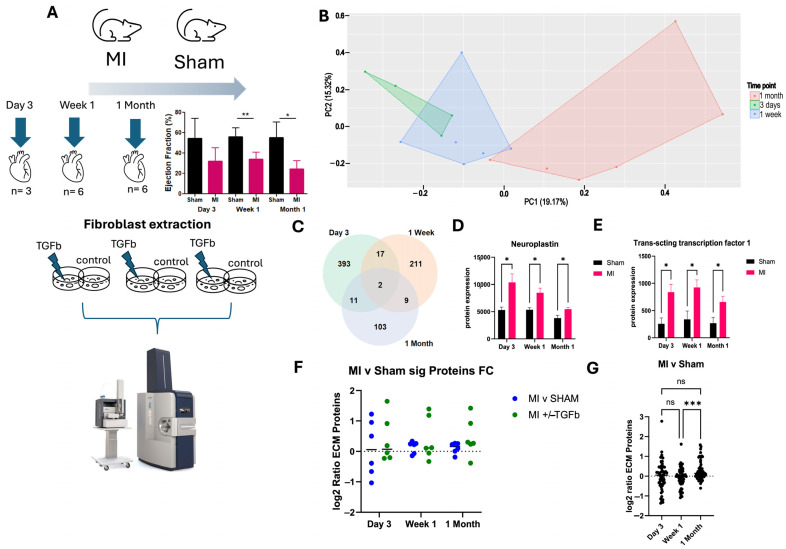
Proteomic characterisation of myocardial infarction experimental mice. Female C57BL6 mice underwent experimental myocardial infarction (MI) by permanent left coronary artery ligation. Mice were sacrificed on day 3, 1 week and 1 month post-surgery. (**A**) Heart tissue from n = 3 (day 3), n = 6 (week 1 and 1 month) mice were used to generate primary fibroblasts, which were treated with the pro-fibrotic cytokine TGF-b or DMSO (control) for 24 h before mass spectrometry analysis using dia-PASEF acquisition. (**B**) Principal component analysis of resulting mass spectrometry data reveals a unique proteomic profile in untreated (control) fibroblasts from MI mice at 1 month. (**C**) Welch’s *t*-test is applied to identify significant protein expression changes between MI and sham cardiac fibroblast cells on day 3, 1 week, and 1 month. (**D**,**E**) Two proteins (Neuroplastin and Trans-transcription factor1) are significantly elevated in MI mice at all time points. (**F**,**G**) In week 1 and 1 month, the average fold changes of extracellular matrix proteins (n = 59) are trending higher in MI cardiac fibroblasts. Bar charts show the mean ± standard error of the mean (SEM). Coloured dot plots (**F**) represent individual samples, and black dots (**G**) represent individual extracellular matrix (ECM) proteins. * *p* < 0.05; ** *p* ≤ 0.01; *** *p* ≤ 0.001; ns, non-significant.

Female C57BL6 mice underwent experimental MI by permanent left coronary artery ligation, which resulted in a reduction in left ventricular ejection fraction (Figure 1A). Mice were sacrificed after 3 days, 1 week and 1 month post-surgery (Figure 1A) to reflect the phases of cardiac wound healing post-infarct, including the inflammatory phase, proliferative phase and the remodelling/maturation phase [6]. Heart tissue from MI and sham experimental mice was used to generate primary fibroblasts. Protein was subsequently extracted from primary fibroblast cells and processed for high throughput liquid chromatography–mass spectrometry, resulting in over 5000 proteins identified across all samples. Principal component analysis revealed some overlap in MI cardiac fibroblast proteome profiles at ‘Day 3’ and ‘Week 1’ post-MI, suggesting an ‘acute’ MI phenotype. Notably, a distinct proteome profile was observed in MI cardiac fibroblasts at 1 month post-MI (Figure 1B). The later time point was considered to be reflective of a more ‘chronic’ MI phenotype. Proteins that are significantly differentially expressed in response to experimental MI cardiac fibroblasts were identified at 3 days (423 proteins), 1 week (239 proteins), and 1 month (125 proteins), compared to sham cardiac fibroblasts. Among them, two differentially expressed proteins were identified between MI and sham cardiac fibroblasts at all three time points (Figure 1C). Both proteins—Neuroplastin and Trans-acting transcription factor 1—were significantly elevated in MI cardiac fibroblasts at all time points (Figure 1D,E). Fifty-nine proteins related to the extracellular matrix (ECM) were measured in the mass spectrometry dataset. Maladaptive ECM proteins were most evident at 1 month post-MI (Figure 1F,G), which indicates that proteome changes at 1 month are related to the established pathological cardiac remodelling that occurs post-MI.

### 2.2. Pathway Dysregulation in Chronic MI Model

**Figure 2:**
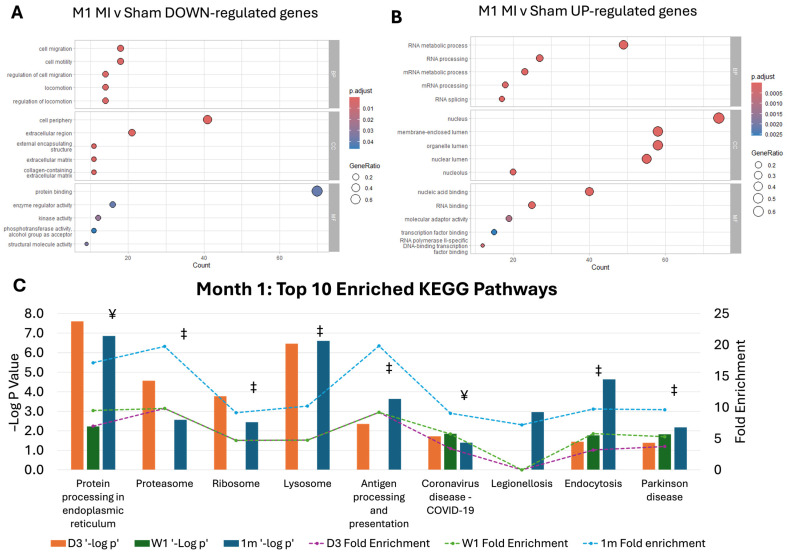
Pathway dysregulation in chronic myocardial infarction experimental model. (**A**,**B**) Gene Ontology analysis reveals biological processes (BP), cellular compartments (CC) and molecular functions (MF) that are more de-regulated by significantly down and upregulated genes 1 month post-myocardial infarction. (**C**) The top most enriched Kyoto Encyclopedia of Genes and Genomes (KEGG) pathways (mouse) associated with protein changes at 1 month post-MI are also de-regulated at day 3 post-MI. ‡ = common up and downregulated genes in D3 and 1m datasets; ¥ = common up and downregulated genes at all time points.

To understand the signature proteome of chronic MI phenotype, we revealed proteins that were significantly differentially expressed between MI and sham cardiac fibroblasts at 1 month post-MI but not at day 3 and week 1 post-MI (Figure 2A,B). These significantly expressed proteins at 1 month post-MI vs. 1 month sham cardiac fibroblasts were significantly associated with a biological process related to cell motility and migration, RNA metabolism and processing (Figure 2A,B). Interestingly, while the top ten enriched KEGG pathways were most significantly associated with 1 month post-MI marked by fold enrichment, the same pattern of fold enrichment was observed for all 10 pathways at day 3 and week 1 time points. The majority of the top ten enriched KEGG pathways associated with proteins de-regulated at 1 month post-MI are also enriched by proteins that are significantly differentially expressed at Day 3. These pathways and associated proteins may, therefore, have a fundamental role in the onset and sustained pathology of MI-associated cardiac dysfunction (Figure 2C).

### 2.3. Effect of TGF-β Treatment on MI Cardiac Fibroblasts

**Figure 3:**
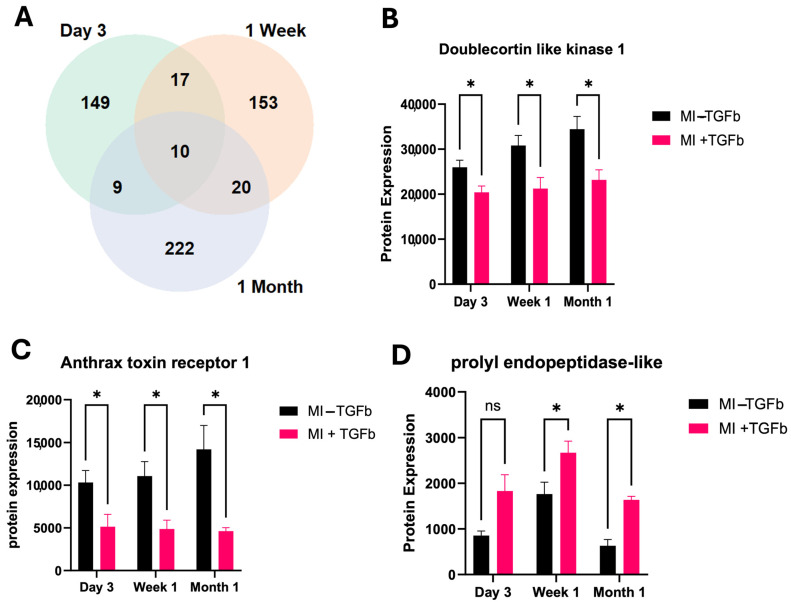
Effect of TGF-β treatment on MI cardiac fibroblasts. (**A**) Welch’s *t*-test is applied to identify significant protein changes as a result of TGF-β treatment of MI cardiac fibroblasts at day 3, 1 week and 1 month time points. (**B**–**D**) Three of the commonly up- and downregulated proteins (Dclk1, Antrx1 and Prepl) at all time points in response to MI. Bar charts show the mean ± standard error of the mean (SEM). * *p* < 0.05; ns, non-significant.

**Figure 4:**
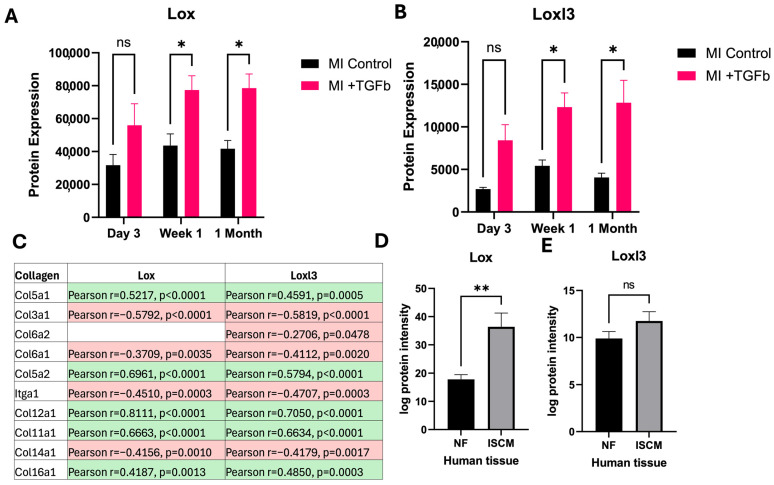
MI sensitises cardiac fibroblasts to pro-fibrotic stimulation by TGF-β. (**A**,**B**) The extracellular matrix proteins Lox and Loxl3 are significantly elevated in MI cardiac fibroblasts after TGF-β treatment. (**C**) Lox and Loxl3 are significantly associated with a number of collagen proteins (red = negative correlation; green = positive correlation). (**D**) Lox and (**E**) Loxl3 are also elevated in human ISCM, compared to patients without clinical heart failure. Bar plots represent the mean ± standard error of the mean (SEM). * *p* < 0.05; ** *p* ≤ 0.01; ns, non-significant.

Comparison of protein expression changes in MI cardiac fibroblasts with and without TGF-β treatment, a pro-fibrotic cytokine at day 3, week 1 and month 1 post-MI revealed a large number of differentially expressed proteins at each time point (Figure 3A). The protein expression of serine/threonine protein kinase, doublecortin-like kinase 1 (Dclk1) and anthrax toxin receptor 1 (Antxr1) significantly decreased in response to TGF-β treatment of MI cardiac fibroblasts at all time points (Figure 3B,C). Furthermore, prolyl endopeptidase-like protein (Prepl) protein expression increased in MI cardiac fibroblasts, compared with sham cardiac fibroblasts in response to TGF-β treatment at week 1 and month 1, and this negatively correlated with left ventricular mass (Figure 3D). Altogether, this underlines an increased sensitivity to pro-fibrotic stimulation by TGF-β in cardiac fibroblasts isolated from MI experimental hearts. Then, we specifically studied proteins related to crosslinked collagen fibres, namely protein-lysine 6-oxidase (Lox) and Lysyl oxidase homolog 3 (Loxl3). Both Lox and Loxl3 were significantly upregulated in MI cardiac fibroblasts in response to TGF-β treatment (Figure 4A,B). These proteins are significantly associated with a number of other collagen proteins, indicating their potential role in the pathogenesis of cardiac fibrosis (Figure 4C). To confirm the pathogenic association with cardiac fibrosis, we found that expression of Lox was significantly elevated in patients with ISCM compared to patients without clinical HF (Figure 4D,E). Expression of Loxl3 was elevated in ISCM cardiac tissue; however, this increase was not determined as statistically significant.

### 2.4. In Silico Validation of Proteomic Changes in Cardiac Fibroblasts

**Figure 5:**
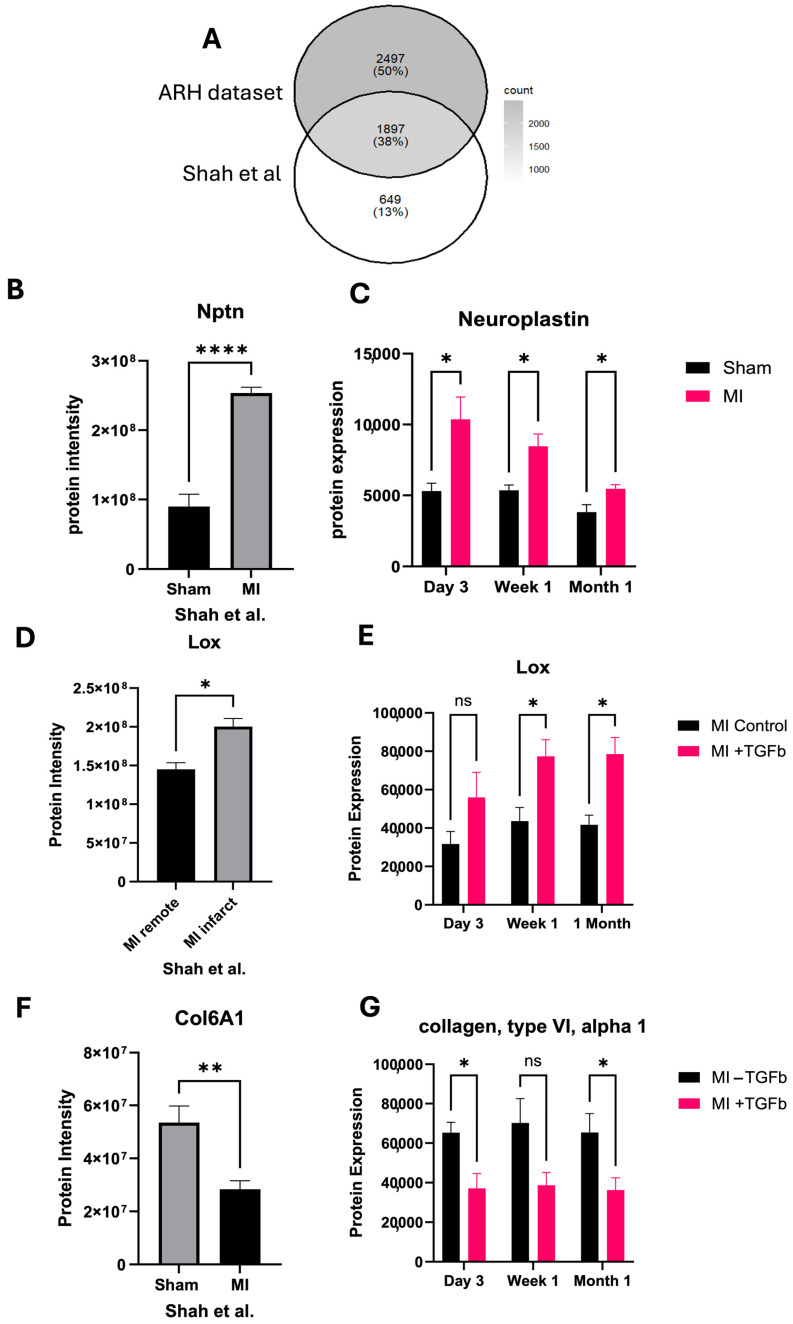
In silico validation of proteomic changes in cardiac fibroblasts. (**A**) Publicly available mass spectrometry data were accessed through ProteomeXchange for validation of observed protein expression changes (Shah et al. [9]). (**B**–**G**) Neuroplastin (Nptn), Lox and collagen type Vi alpha 1 (Col6A1) were verified as being significantly associated with MI. Bar plots represent the mean ± standard error of the mean (SEM). * *p* < 0.05; ** *p* ≤ 0.01; **** *p* ≤ 0.0001; ns, non-significant.

Mass spectrometry data generated previously by Shah et al. [9] was used to validate signature proteomic changes observed in this study. With a similar approach to assessing cardiac fibroblasts isolated from MI heart mice, Shah et al. [9] performed mass spectrometry analysis of cardiac fibroblasts generated from the hearts of mice subject to experimental MI and sacrificed after 7 days. Thirty-eight percent of proteins identified in our dataset were also identified in Shah et al.’s dataset [9] (Figure 5A). The protein Neuroplastin, which was significantly elevated in MI cardiac fibroblasts at all time points in our study, was also significantly associated with MI in the Shah et al.’s dataset [9] (Figure 5B,C). Furthermore, Lox was found to be substantially increased in cardiac fibroblasts generated from the site of MI infarct, compared to remote regions (Figure 5D,E). This underlines further evidence of a potential fundamental role of Lox in driving the pro-fibrotic response of MI-injured cardiac fibroblasts. Protein collagen, type VI, alpha 1 (Col6a1) was significantly downregulated in MI cardiac fibroblasts in response to TGF-β treatment; this is also significantly downregulated in response to MI in the Shah et al.’s dataset [9] (Figure 5F,G).

## 3. Discussion

Over the last decade, HF prevalence has significantly increased. Although prompt urgent revascularisation and effective treatment strategies have greatly reduced acute MI mortality, IHD continues to be a leading cause of HF [11,12]. After a pathogenic myocardial injury, the heart undergoes a complex process of structural and functional remodelling through several processes, including inflammatory and fibrotic responses [13]. While cardiac troponin is the most commonly used cardiac enzyme for MI diagnosis, it does not peak until 18 to 24 h after symptom onset and can be detected in the blood for up to 14 days [14]. However, detecting biomarkers related to myocardial damage at a more extended time point might be challenging. Therefore, this is an opportunity to provide additional markers that have been sought in order to provide better risk stratification in both acute and chronic phases.

A previous study showed increasing numbers of differentially expressed proteins responding to experimental MI at sequential time points (10 min, 1 h, 6 h, 24 h, and 72 h) [2]. Therefore, we provide important data on pathogenic remodelling within cardiac fibroblasts beyond the early inflammatory and proliferative phases until the remodelling/maturation phase. In our study, a distinct proteome signature between the ‘acute’ MI phase (3 days and 1 week) and the ‘chronic’ MI phase (1 month) is evident. Moreover, the current study reveals that maladaptive ECM protein expression was most pronounced 1 month after the MI event. These results support the concept of ‘transition from infarction to remodelling’ [13,15]. After acute MI, pressure and volume overload increase wall stress, impairing left ventricular function, and immune cell infiltration triggers scar formation and cardiomyocyte loss. In the chronic phase, persistent inflammation and other factors drive extracellular matrix expansion and ongoing remodelling [13,16]. Interestingly, we found neuroplastin and trans-acting transcription factor 1 were increased in MI cardiac fibroblast at all timeframes. Neuroplastin is upregulated under ER stress and can induce inflammation via NF-kB activation [17], while trans-acting transcription factor 1 (also known as Sp1) is associated with MI and cardiac hypertrophy [18,19].

It is becoming increasingly understood that activation of fibroblasts in the context of cardiac injury leads to the establishment of a persistent pro-fibrotic cellular phenotype enhancing cardiac fibrosis [20,21]. To understand this further, we studied isolated cardiac fibroblasts from both MI and sham environments and their response to pro-fibrotic cytokine, TGF-β. The serine/threonine protein kinases are linked to multiple cardiovascular diseases such as ischemia-reperfusion injury, HF, and MI [22]. However, the specific role of doublecortin-like kinase 1 (Dclk1) in cardiovascular diseases is limited. It has been reported that specific deletion of Dclk1in macrophages has demonstrated a reduction in cardiac hypertrophy, myocardial fibrosis and atherosclerotic plaques [23,24]. An additional study has provided evidence that reducing Dclk1 in the context of diabetic cardiomyopathy through genetic knockout or inhibitors can reduce cardiac fibrosis [25]. Translating these studies to our findings would suggest that decreased Dclk1 in response to TGF-β treatment in isolated MI fibroblasts could be a mechanism to balance and modulate a pro-fibrotic response. Further functional studies of Dclk1 in MI are required to elucidate its true role in this context.

Anthrax toxin receptor 1 deficiency promotes fibroblast senescence and links to the ECM and cell-matrix adhesion process [26,27]. Moreover, prolyl endopeptidase-like protein was increased in our isolated fibroblasts treated with TGF-β from MI mice, and this protein primarily involves mitochondrial function, and deficiency in prolyl endopeptidase-like protein can lead to mitochondrial dysfunction, which may impact cardiac health and complications [28,29]. However, our isolated cardiac fibroblast treated with TGF-β increased in prolyl endopeptidase-like protein, suggesting multifunctional roles in pathogenic MI. We notably found that the protein levels of Lox and Loxl3 were elevated at week 1 and 1 month in isolated cardiac fibroblasts treated with TGF-β from MI mice, and these proteins centrally involve crosslinking and stabilising collagen and elastin fibres, including cardiac remodelling [30,31]. Increased expression of Lox was subsequently validated in both clinical samples from patients with ISCM and in cardiac fibroblasts from the site of MI infarct [9]. Changes in Lox and Loxl3 are associated with different collagen protein expressions. Therefore, these highlight the important roles of Lox and Loxl3 in pathogenic cardiac remodelling. Collagen cross-linking occurs through two distinct pathways: an enzymatic process facilitated by enzymes from the transglutaminase or LOX families and a nonenzymatic (promoted by advanced glycation end-products; AGEs) process [32,33]. Evidence from human and animal studies suggests that dysregulated LOX/LOXL isoenzyme function or expression has been linked to cardiovascular diseases [32,34]. In a prospective study, elevated circulating soluble LOX-1 is associated with the risk for first-time MI [35]. Furthermore, the upregulation of LOX isoforms (LOX and LOXL1–4), accompanied by a significant accumulation of mature collagen fibres within the infarcted region, was confirmed in experimental MI mice [36]. Taken together, these results, along with our findings, highlight potential roles for LOX and LOXL modulation as biomarkers related to cardiac remodelling following MI and as potential therapeutic targets for post-MI recovery. Additionally, we found that Col6a1 was significantly decreased in our dataset (isolated MI cardiac fibroblasts stimulated by TGF-β vs. isolated sham cardiac fibroblasts stimulated by TGF-β) and in the external dataset from Shah et al. [9]. Col6 is a nonfibrillar collagen highly expressed in developing and adult hearts [37]. Previous research has reported that following infarction, Col6 was elevated in vivo and induced myofibroblast differentiation [38,39]. Col6a deficiency can be protective by limiting the extent of fibrosis and scar formation, potentially improving cardiac function after the injury [40]. Therefore, further experimental studies are necessary to elucidate the specific roles of Col6a1 and to provide mechanistic insights that distinguish Col6a1 from other collagen subtypes. From a clinical perspective, noninvasive techniques such as speckle tracking echocardiography (STE) may help to identify myocardial areas with reduced deformation due to myocardial fibrosis. Lower myocardial deformation in cardiac chambers is associated with a greater extent of myocardial fibrosis within the heart walls. STE analysis offers clinicians a noninvasive method to detect areas of myocardial fibrosis in both coronary artery disease (CAD) [41] and non-CAD [42] contexts. The inclusion of STE analysis in translational studies would enhance the phenotyping of cardiac remodelling.

Cardiac fibroblasts undergo temporal phenotypic changes throughout the wound healing response, including post-MI. Cardiac fibroblast activation and how they respond to external signals may vary in the context of MI, as indicated by the differential response of MI-derived fibroblasts to TGFβ in our study. Cardiac fibroblasts exhibit different cellular phenotypes and physiological roles at varying times post-MI [43,44,45]. These changes include the inflammatory phase with activation of various cytokines (e.g., IL-1 β, IL6) and chemokines (e.g., CXCL-8, CCL-2) [46,47,48], the proliferative phase and maturation phase, which are important processes to induce transdifferentiation of fibroblasts into myofibroblasts and generation of ECM proteins through anti-inflammatory cytokines (e.g., IL-10) and pro-fibrotic factors (e.g., TGF-β1) [49,50,51]. Ultimately, our findings support the understanding of the post-MI fibroblast response and may reveal insights into cardiac remodelling and identify novel targets to improve treatments.

Altogether, our study with cross-sectional follow-up following an MI event provides protein signatures related to myocardium damage and underlines the unique response of cardiac fibroblasts to pro-fibrotic cytokines. The differential proteomic response to pro-fibrotic stimuli in those cells previously exposed to a post-MI microenvironment poses potential therapeutic targets to alleviate aberrant cardiac remodelling and HF development. Our study contains limitations. The murine model of MI was conducted using female mice, and the human dataset within our research uses only males. It is important to note that sex differences, which are well known in the context of myocardial remodelling, such as hormone regulation and immune/inflammatory regulation [52], could potentially influence the translation of research findings into clinical studies. An example of this has been demonstrated where estrogen supplementation in non-castrated male mice led to improved cardiac function and attenuated cardiac remodelling after MI [53]. To overcome this hurdle, we validated our results using the dataset from Shah et al. [9], which was generated using both male and female mice. This suggests that changes in the candidate proteins identified in this study, which are associated with myocardial injury, occur independently of sex. While our study highlights the potential role of Lox and Loxl3 in cardiac fibroblasts and cardiac remodelling post-MI, we did not investigate the experimental impact of these candidate proteins in this study. Modulation of these extracellular proteins using genetic or pharmacological approaches, specifically in fibroblasts, may help elucidate their precise roles and impact in ischaemic cardiomyopathy.

## 4. Materials and Methods

### 4.1. Human Data and Analysis

Left ventricular (LV) tissue samples were collected from male patients who underwent orthotropic cardiac transplantation for ischaemic cardiomyopathy (ISCM) (n = 9). Matched control patients were non-failing hearts (n = 9) who died of noncardiac causes. The Institutional Review Board at the Cleveland Clinic provided ethical permission for the use of tissue and data collecting. Descriptions of patient demographics and clinical features were previously reported [54]. Proteomic sample preparation and analysis of LV tissue samples were previously described [55]. Briefly, using a timsTOF Pro (Bruker, Billerica, MA, USA) quadrupole time-of-flight mass spectrometer, which integrates trapped ion mobility separations coupled online via a Captivespray electrospray source (Bruker) to a nanoElute (Bruker) nanoflow liquid chromatography system, mass spectrometry (MS) proteomics data were obtained. Then, Spectronaut software version 18 was utilised to generate the spectral library, and an independent Student’s *t*-test was applied to obtain differences between groups with FDR correction. All *p* values < 0.05 were significant changes [55].

### 4.2. In Vivo Model of Experimental MI

Female C57Bl/6 mice (8–10 week old) were obtained from Charles River UK (Harlow, UK) and underwent experimental MI by permanent left coronary artery ligation, as described previously [56]. Briefly, a 7-0 suture was passed under the left anterior descending (LAD) coronary artery and permanently tied using a single interrupted suture. Sham mice underwent the full surgical procedure, except the 7-0 suture was not tied off after passing under the LAD. Mice were randomly selected to undergo sham or MI surgery. After successful surgical induction of MI, mice were allocated to their designated time points and sacrificed at 3 days, 1 week and 1 month post-surgery. Surgical-matched sham mice from the same day were used as the control groups at each time point. This experimental MI model was previously well-established for MI characteristics in a previous study [57]. All animal work was conducted under guidelines established by Directive 2010/63/EU of the European Parliament on the protection of animals used for scientific purposes and UK Home Office regulations. All experimental protocols were approved by the Biological Services Unit at Queen’s University Belfast.

### 4.3. Echocardiography Data Acquisition and Data Analysis

After 3 days, 7 days and 28 days post-op, mice were anaesthetised with 1.5% isoflurane in oxygen and imaged in the supine position using a Vevo 770 ultrasound system with high-frequency 45 MHz RMV707B scanhead (VisualSonics, Toronto, ON, Canada). Core temperature was maintained at 37 °C, and heart rates were kept consistent between experimental groups (400–500 BPM). Electrocardiogram (ECG) was derived using Limb electrodes. Standard 2D echocardiographic images were obtained from the parasternal long-axis view for assessment of left ventricular dimensions and function.

### 4.4. Cardiac Fibroblast Isolation and Culture

After cardiac imaging, animals were sacrificed, and cardiac fibroblasts were isolated from the entire left ventricle as previously described [58]. Briefly, excised cardiac tissue was rinsed, minced and then digested by collagenase II (600 U/mL, 17101015; Gibco™, Waltham, MA, USA) and DNase I solution (60 U/mL, 18047019; Invitrogen™, Waltham, MA, USA) in Hanks-buffered saline solution (14025092; Gibco™, Waltham, MA, USA). After 1 h incubation at 37 °C, with mechanical dissociation applied every 15 min, the entire cell suspension was filtered through a 30 µm cell strainer, centrifuged, and resuspended in Dulbecco’s Modified Eagle Medium (DMEM; 11965092; Gibco™, Waltham, MA, USA) supplemented with 10% fetal bovine serum (FBS; Gibco™, Waltham, MA, USA) and 1× antibiotic–antimycotic solution (15240-062; Gibco™, Waltham, MA, USA). Cells were subsequently transferred to T25 flasks and incubated under standard cell culture conditions (37 °C; 5% CO_2_). The cells from P1 were used for the experiments. To assess pathogenic response within isolated cardiac fibroblasts from MI and sham mice, isolated cardiac fibroblasts in serum-free media were treated with TGF-β (5 µg/mL (R&D Systems, Minneapolis, MN, USA, 7754-BH-005/C)) for 24 h. Cell pellets were collected and stored at 80 °C until further analysis. 

### 4.5. Sample Preparation for Mass Spectrometry Analysis

Primary cardiac fibroblast cells were lysed in Urea lysis buffer (8M Urea, 0.1M Tris-Cl pH 8.0). Protein samples were quantified using the bicinchoninic acid (BCA) assay (23227; Thermo Scientific™, Waltham, MA, USA). Twenty-five μg of protein was denatured in a final concentration of 10 mM 1,4-dithiothreitol (DTT) (Roche, Mannheim, Germany) and alkylated with 14 mM iodoacetamide (IAA) (I1149, Sigma-Aldrich, Gillingham, UK). Protein was digested with a 1:50 protein–enzyme ratio of Trypsin (V5111; Promega, Madison, WI, USA). Peptides were dried down under a vacuum, resuspended in 1% trifluoroacetic acid (TFA) (302031, Sigma-Aldrich, Gillingham, UK) and then de-salted on C18-packed stage-tip columns. Briefly, C18 stage-tips were activated with 50 μL of 50% acetonitrile (AcN; 34851; Sigma-Aldrich, Gillingham, UK)/0.1% TFA. The stage-tips were washed with 1% TFA before adding ~8 µg of peptide in 1% TFA. After a further two wash steps with 1% TFA, the peptide was eluted from the stage-tip in 25 μL of 50% AcN/0.1% TFA. All digested samples were pooled based on experimental groups and fractionated using the Pierce™ High-pH Reversed-Phase Peptide Fractionation Kit (84868; Thermo Scientific™, Waltham, MA, USA), as per the manufacturer’s instructions. Prior to mass spectrometry analysis, peptide samples were dried down under a vacuum, re-constituted in 0.1% formic acid (F0507; Sigma-Aldrich, Gillingham, UK) and loaded onto EvoTips™ (Odense, Denmark), as per manufacturer’s instructions. 

### 4.6. Mass Spectrometry Analysis

All samples were analysed as part of one experimental run on a timsTof Pro mass spectrometer (Bruker Daltonics, Billerica, MA, USA) connected to an Evosep One liquid chromatography system (EvoSep BioSystems, Odense, Denmark). A reversed-phase C18 Endurance column using the 30 SPD method was used for peptide separation. Data-dependent acquisition mode (DDA) was used for the analysis of high pH-reversed phase fractionated sample pools to generate data for the spectral library. Data independent acquisition mode (DIA) was used to analyse individual samples. Trapped ion mobility spectrometry (TIMS) mode was used for data acquisition. Parallel accumulation serial fragmentation (PASEF) was used to select trapped ions for ms/ms. Regular intervals throughout the pooled sample digests were analysed as quality control.

### 4.7. Data Analysis

Fragpipe (version 22.0) software (https://github.com/Nesvilab/FragPipe accessed on 11 August 2024) was used to generate spectral library files from the acquired DDA data. DIA-NN (version 1.9.2) software (https://github.com/vdemichev/DiaNN accessed on 14 August 2024) was used to process raw (.d) diaPASEF data files for a spectral library building, protein identification and quantification. Data processing was performed using R (version 4.4.1) software. Raw data were log-transformed, and proteins with >70% missing values across all samples were removed. Missing data for the remaining protein were imputed by sampling values from a normal distribution. To compare means between two groups, Welch’s *t*-test was employed. All tests were two-tailed with a predefined significance level of *p*  <  0.05. Pathway analysis was carried out using clusterProfiler version 4.12.6 [59] based on ontology and KEGG (Kyoto Encyclopedia of Genes and Genomes) databases. Statistical analyses were performed using GraphPad Prism 9 (GraphPad Software, San Diego, CA, USA).

## 5. Conclusions

These current findings open avenues for drug repurposing and the design of novel therapeutic strategies aimed at modulating key proteins involved in fibrosis progression after myocardial injury. By identifying specific proteomic alterations associated with maladaptive remodelling, our study also paves the way for the development of biomarker-driven precision medicine approaches. These biomarkers may serve as indicators of post-MI progression, predictors of therapeutic response, or even surrogate endpoints in clinical studies.

## Data Availability

The data that support the findings of this study are available from the corresponding author upon reasonable request.

## Abbreviations

The following abbreviations are used in this manuscript:

IHD	ischaemic heart disease
HF	heart failure
TGF-β	Transcription Growth Factor Beta
LC-MS/MS	Liquid Chromatography Tandem Mass Spectrometry
ISCM	ischaemic cardiomyopathy
NF	Non-failure
MI	myocardial infarction
ECM	extracellular matrix
FDA	Food and Drug Administration
LV	left ventricular
MS	mass spectrometry
LAD	left anterior descending
UK	United Kingdom
BPM	Beats per minute
ECG	Electrocardiogram
DMEM	Dulbecco’s Modified Eagle Medium
FBS	fetal bovine serum
BCA	Bicinchoninic acid assay
DTT	1.4-dithiothreitol
IAA	iodoacetamide
TFA	trifluoroacetic acid
AcN	acetonitrile
DDA	Data dependent acquisition
DIA	Data independent acquisition
TIMS	trapped ion mobility spectrometry
PASEF	Parallel accumulation serial fragmentation
KEGG	Kyoto Encyclopedia of Genes and Genomes
RNA	Ribonucleic acid
Dclk1	doublecortin-like kinase 1
Antxr1	anthrax toxin receptor 1
Prepl	prolyl endopeptidase-like protein
Lox	protein-lysine 6-oxidase
Loxl3	Lysyl oxidase homolog 3
Col6a1	Protein collagen, type VI, alpha 1
ER	Endoplasmic reticulum
NF-kB	Nuclear factor kappa-light-chain-enhancer of activated B cells
